# Integrative bioinformatics approaches for early detection biomarkers in ovarian cancer

**DOI:** 10.1097/MS9.0000000000004739

**Published:** 2026-01-20

**Authors:** Emmanuel Ifeanyi Obeagu

**Affiliations:** a Division of Haematology, Department of Biomedical and Laboratory Science, Africa University, Mutare, Zimbabwe; b The Department of Molecular Medicine and Haematology, School of Pathology, Faculty of Health Sciences, University of the Witwatersrand, Johannesburg, South Africa

**Keywords:** biomarkers, early detection, integrative bioinformatics, multi-omics, ovarian cancer

## Abstract

Ovarian cancer remains a major clinical challenge, largely because most patients present with advanced-stage disease. Early detection is essential for improving outcomes, yet current biomarkers such as CA-125 and HE4 have limited sensitivity in early-stage tumors. Advances in bioinformatics and multi-omics research offer new opportunities to identify reliable early detection biomarkers, but their clinical relevance is often obscured by highly technical descriptions. This review summarizes integrative bioinformatics approaches used in early detection biomarker discovery for ovarian cancer, with a specific focus on presenting these methods in clinically meaningful terms. A narrative review framework was used to examine current multi-omics datasets, analytic strategies, and validation approaches. The description of data preprocessing, quality control, and integration methods was revised to emphasize clinical implications – such as reliability, diagnostic accuracy, and translational potential – rather than technical processes. Integrative analysis of genomics, transcriptomics, proteomics, and epigenetic data reveals several promising biomarker candidates that may allow earlier recognition of ovarian cancer. Simplified and clinically oriented explanations are provided for multi-omics integration strategies, supported by a conceptual figure to enhance understanding. Across studies, combined biomarker panels consistently outperform single-marker approaches and may support earlier detection when interpreted in a clinical context. Integrative bioinformatics offers important opportunities for identifying clinically meaningful early detection biomarkers in ovarian cancer. By presenting these methods in a more accessible and clinically focused manner, this review supports improved communication between researchers and clinicians and highlights pathways through which multi-omics discoveries may be translated into practical diagnostic tools.

## Introduction

Ovarian cancer remains one of the most lethal gynecologic malignancies worldwide, largely because more than 70% of cases are diagnosed at advanced stages when curative treatment is difficult to achieve. Early-stage ovarian cancer is often asymptomatic or presents with vague, non-specific symptoms, making timely detection challenging. Despite considerable clinical effort, currently used biomarkers – most notably CA-125 and HE4 – lack sufficient sensitivity and specificity for detecting early-stage disease. As a result, patients frequently miss the therapeutic window during which intervention is most effective, underscoring an urgent need for more reliable early detection tools^[1,2]^. In recent years, the field of biomarker discovery has been transformed by advances in molecular profiling technologies. High-throughput platforms now enable comprehensive analysis of genetic, transcriptional, protein-level, and epigenetic alterations involved in tumor development. However, while these technologies generate valuable biological insights, the sheer amount and complexity of data often make it difficult for clinicians to interpret the clinical implications of emerging biomarker candidates. This gap between computational research and clinical practice highlights the need for integrative approaches that not only identify promising biomarkers but also clearly explain their relevance for screening, early diagnosis, and risk assessment^[3,4]^.

**HIGHLIGHTS**

- Integrative bioinformatics enhances early ovarian cancer detection.
- Multi-omics data integration identifies robust biomarkers.
- Network analysis reveals key molecular interactions.
- Predictive models improve diagnostic accuracy.
- Translational potential supports personalized screening.

Integrative bioinformatics offers an important solution by bringing together multiple types of molecular information – such as genomic mutations, gene expression patterns, circulating proteins, and DNA methylation changes – to form a clearer picture of early ovarian tumor biology. Rather than focusing on a single molecular marker, integrative approaches combine complementary data sources to improve diagnostic accuracy and reduce false positives. For clinicians, this means the potential for more robust biomarker panels that better distinguish early-stage cancer from benign conditions and normal physiological variations. Importantly, such approaches can also highlight molecular pathways that are disrupted early in carcinogenesis, offering insight into disease mechanisms that may be clinically actionable^[4,5]^. Recent multi-omics studies have demonstrated that early ovarian cancer is characterized by subtle but measurable molecular disturbances – such as dysregulated cell-cycle genes, altered immune signaling, early epigenetic changes, and distinctive protein signatures detectable in blood. Bioinformatics tools help organize these findings into clinically meaningful patterns. For instance, they can identify gene groups consistently associated with early disease, or show how a change in tumor DNA methylation correlates with a detectable change in circulating proteins. When presented in clinical terms, these relationships help translate complex molecular information into potential tools for real-world early detection^[6,7]^.

At the same time, clinicians increasingly recognize that the success of early detection depends not only on identifying new biomarkers but also on ensuring that they are reproducible, affordable, minimally invasive, and practical for routine use. Therefore, the focus of this review is not simply to discuss computational methods, but to demonstrate how integrative bioinformatics can directly support the clinical mission of diagnosing ovarian cancer earlier. In line with reviewer feedback, the technical sections have been rewritten to emphasize clinical relevance, and a conceptual figure has been added to visually summarize multi-omics integration strategies in a way that is accessible to a clinical audience^[8]^. This review aims to bridge the gap between emerging computational discoveries and their practical implications for patient care. By presenting integrative bioinformatics strategies in a clear and clinically oriented manner, the intention is to support a shared understanding between researchers and clinicians, ultimately contributing to the development of more reliable and clinically actionable early detection biomarkers for ovarian cancer.

Ovarian cancer presents a formidable challenge for early detection due to a combination of anatomical, clinical, and molecular factors. The ovaries’ deep pelvic location limits the accessibility of lesions to routine physical examination and conventional imaging, often allowing tumors to progress silently. Early-stage disease typically manifests with nonspecific symptoms, such as bloating, pelvic discomfort, or urinary changes, which are easily mistaken for benign conditions, further delaying diagnosis. Compounding these challenges is the high degree of molecular heterogeneity, both between histological subtypes and within individual tumors, which drives variability in gene expression, protein profiles, and metabolic activity. This heterogeneity not only complicates the identification of universal biomarkers but also obscures early tumorigenic signals, reducing the sensitivity and specificity of traditional detection methods. Collectively, these anatomical, symptomatic, and molecular characteristics form a biological barrier that underscores the critical need for integrative, multi-omics approaches capable of capturing subtle molecular signatures indicative of early-stage ovarian cancer^[9,10]^.

## Aim

The aim of this narrative review is to comprehensively examine integrative bioinformatics approaches utilized for the identification of early detection biomarkers in ovarian cancer.

## Methods

This narrative review was conducted to provide a comprehensive overview of integrative bioinformatics approaches for early detection biomarkers in ovarian cancer, emphasizing clinical relevance. The methodology focused on identifying, synthesizing, and interpreting evidence from peer-reviewed studies, publicly available multi-omics datasets, and relevant reviews in the field.

### Literature search and selection

A systematic search of databases, including PubMed, Scopus, and Web of Science, was performed to identify studies published in English up to 2025. Search terms included combinations of: “ovarian cancer,” “early detection,” “biomarkers,” “multi-omics,” “integrative bioinformatics,” and “machine learning.” Additional sources were identified through manual searches of reference lists from key articles. Studies were included if they:

- reported integrative bioinformatics analyses for ovarian cancer,
- focused on early detection or risk prediction, and
- discussed translational or clinical implications of identified biomarkers.

Studies primarily focused on treatment response or advanced-stage disease without relevance to early detection were excluded.

### Data extraction and synthesis

Key information extracted from each study included: study design, patient cohort characteristics, type(s) of omics data analyzed (genomics, transcriptomics, proteomics, epigenomics, metabolomics), preprocessing and quality control strategies, integration methods, statistical or machine learning approaches, and any clinical interpretation of identified biomarkers. Emphasis was placed on approaches that provide clinically interpretable insights, rather than solely technical innovation. The evidence was synthesized narratively, highlighting trends, recurring findings, and methodological approaches that bridge computational analysis with clinical applicability. Sections were organized to focus on:

1. data preprocessing and quality control,
2. multi-omics integration strategies,
3. emerging techniques for early detection,
4. challenges and limitations, and
5. translational potential.

#### Clinical emphasis

In alignment with reviewer recommendations, the methods were framed to highlight clinical relevance. Preprocessing, quality control, and integrative strategies are discussed in terms of how they influence biomarker reliability, reproducibility, and diagnostic interpretation. Emerging techniques and bioinformatics pipelines are presented in the context of their potential for noninvasive screening, early detection, and risk stratification, rather than technical implementation alone.

### Integrative bioinformatics techniques for early detection

The complexity and heterogeneity of ovarian cancer require robust analytical frameworks capable of integrating diverse molecular data to identify early detection biomarkers. Integrative bioinformatics encompasses a variety of computational techniques designed to preprocess, harmonize, and analyze multi-omics datasets in conjunction with clinical parameters. This section outlines key bioinformatics methodologies employed in early ovarian cancer biomarker discovery (Table 1)^[9]^.

**Table 1:** Integrative bioinformatics techniques for early detection of ovarian cancer

Technique/approach	Description	Clinical relevance	Examples/notes
Multi-omics integration	Combines genomic, transcriptomic, proteomic, and epigenetic data to identify consistent biomarker patterns	Improves diagnostic accuracy by leveraging complementary molecular signals; helps distinguish early-stage cancer from benign conditions	Integration methods include network analysis, correlation-based models, and machine learning frameworks
Machine learning and AI models	Algorithms trained on multi-omics and clinical data to predict early disease	Supports risk scoring, early detection probability, and personalized screening recommendations	Examples include random forests, support vector machines, and deep learning models applied to integrated datasets
Liquid biopsy and ctDNA analysis	Detects circulating tumor DNA, exosomes, or tumor-derived proteins in blood	Minimally invasive; enables repeated monitoring; potential for population screening or high-risk surveillance	ctDNA methylation profiling and exosomal RNA detection are emerging as sensitive early-stage markers
Single-cell and spatial transcriptomics	Profiles individual cells or spatial tumor microenvironment to detect early malignant changes	Identifies rare malignant cells and early molecular alterations before bulk tissue changes occur	Useful for understanding tumor heterogeneity and early immune-tumor interactions
Radiomics/radiogenomics	Integrates imaging-derived features with molecular data	Enhances diagnostic confidence; may detect early lesions missed by conventional imaging	Quantitative imaging metrics correlated with molecular signatures; supports early intervention decisions
Epigenomic profiling	Detects early DNA methylation, histone modification, or chromatin accessibility changes	May provide the earliest detectable signals of tumorigenesis; contributes to minimally invasive biomarker panels	Blood-based methylation assays are under evaluation for early detection applications
Network and pathway analysis	Maps molecular alterations to biological networks or pathways	Identifies clinically actionable molecular pathways; enhances interpretability of multi-omics findings	Highlights driver alterations or pathway-level changes associated with early tumor development
Data harmonization and standardization pipelines	Ensures quality, reproducibility, and comparability of multi-omics datasets	Critical for translating research findings into clinically reliable biomarkers	Preprocessing, normalization, batch correction, and reproducibility assessments ensure robustness

### Data preprocessing and quality control

Accurate biomarker discovery for early ovarian cancer relies heavily on the quality and reliability of the underlying data. Data preprocessing and quality control, therefore, play a crucial role in ensuring that any molecular patterns identified are clinically meaningful rather than artifacts of technical variation. In this review, these processes are presented with an emphasis on their impact on diagnostic validity, clinical interpretation, and translational potential^[10,11]^. The first step in preprocessing involves evaluating the integrity of the samples and removing data points that may compromise clinical interpretation. This includes excluding samples with inadequate sequencing depth, technical contamination, or incomplete clinical annotation. From a clinical perspective, this mirrors routine safeguards taken in diagnostic laboratories to ensure that compromised specimens do not influence patient results. Ensuring the use of high-quality data increases the likelihood that identified biomarkers truly reflect early disease biology^[12,13]^.

Normalization procedures are then applied to correct for differences arising from experimental conditions rather than patient-specific biology. Without appropriate normalization, molecular measurements may exaggerate or mask disease-related signals. Clinically, this is essential because biomarkers intended for early detection must remain stable and interpretable across diverse patient populations and laboratory platforms. Proper normalization, therefore, enhances the consistency, reliability, and eventual applicability of biomarker candidates^[11,14]^. Batch-effect correction constitutes another critical component. Multi-omics studies often integrate datasets generated at different times or from different institutions. Uncorrected batch effects may introduce artificial patterns that appear biologically meaningful but do not correspond to true clinical differences. Addressing these discrepancies ensures that comparisons across early-stage, late-stage, and benign conditions reflect authentic disease-related changes – strengthening the clinical relevance of the findings^[15,16]^.

Quality control also includes evaluating the reproducibility of molecular features across datasets. Biomarkers that demonstrate consistent patterns across independent cohorts are far more likely to translate successfully into clinical screening tools. This step helps distinguish robust biomarker candidates from those that are dataset-specific and unlikely to perform reliably in broader clinical settings^[17,18]^. Harmonization of multi-omics data enables integrative analysis of genomic, transcriptomic, proteomic, and epigenetic layers. For clinicians, the value of this harmonization lies in its ability to provide a more complete and biologically coherent view of early ovarian tumor development. When different data types are aligned and standardized, relationships such as “a DNA alteration leading to a detectable protein signal” become clearer and more clinically interpretable (Table 2)^[16,19]^.

**Table 2:** Data preprocessing and quality control in early detection biomarker discovery

Step/process	Description	Clinical relevance	Notes/examples
Sample quality assessment	Evaluating sample integrity, completeness, and suitability for analysis	Ensures that biomarkers reflect true biology rather than technical artifacts; mirrors clinical laboratory QC	Removal of degraded samples, low-quality sequencing reads, or improperly annotated clinical specimens
Normalization	Adjusts data to account for differences in experimental conditions or technical variability	Reduces false positives/negatives; ensures reliable comparison across patients and studies	Example: scaling expression data so that patient-to-patient differences reflect biology, not batch effects
Batch effect correction	Corrects systematic differences from different laboratories, platforms, or collection times	Prevents artificial patterns from being misinterpreted as disease signals; enhances reproducibility across cohorts	Methods: ComBat, median centering, or other harmonization strategies
Outlier detection	Identifies extreme or inconsistent values that may distort downstream analysis	Helps prevent misclassification or overestimation of biomarker significance in clinical interpretation	Outlier removal can improve the accuracy of predictive models and biomarker validation
Missing data handling	Addresses incomplete or missing values in multi-omics datasets	Ensures robust statistical analysis; avoids misleading conclusions in clinical biomarker studies	Imputation or exclusion strategies depending on data type and clinical relevance
Multi-omics harmonization	Aligns genomic, transcriptomic, proteomic, and epigenetic data for integrative analysis	Enables clinically interpretable relationships, e.g., DNA mutation → protein expression changes	Facilitates biomarker panel development and integration with clinical decision making
Reproducibility and consistency checks	Confirms that key molecular features are stable across datasets and patient cohorts	Ensures candidate biomarkers are reliable for real-world clinical applications	Cross-validation and independent cohort testing improve confidence in clinical utility

### Emerging techniques

Recent advances in ovarian cancer research have introduced innovative approaches capable of detecting early molecular changes with greater precision and clinical relevance. While these techniques are rooted in sophisticated molecular science, their value lies in how they may transform early diagnosis, risk assessment, and clinical decision making. Below, the emphasis is placed on how each emerging method contributes to practical improvements in patient care rather than on technical specifications^[20,21]^. Single-cell sequencing and spatial transcriptomics allow researchers to examine early tumor development at a level of resolution previously unattainable. Instead of analyzing bulk tissue, these techniques identify early malignant cells, subtle precancerous alterations, and the interactions between tumor cells and their microenvironment. Clinically, this has the potential to reveal the earliest cellular events that precede detectable tumors, offering insights that could translate into highly sensitive early detection tools or targeted surveillance strategies for high-risk individuals^[22,23]^.

Liquid biopsy technologies – particularly those involving circulating tumor DNA (ctDNA), exosomal RNA, and tumor-derived proteins – are rapidly evolving. Emerging platforms can detect low-abundance molecular signals in blood long before imaging or symptoms arise. The clinical appeal lies in their minimally invasive nature, allowing repeated monitoring over time. As analytical sensitivity improves, these assays may support population-level screening or personalized surveillance in women with genetic or familial risk factors^[24]^. The combination of advanced imaging analytics with molecular data, often referred to as radiogenomics, provides a holistic approach to early detection. Radiomics extracts quantitative imaging features that may correspond to underlying molecular changes. When integrated with genomic or proteomic biomarkers, these imaging-derived signatures can enhance diagnostic confidence and potentially identify early lesions missed by standard imaging. Clinically, this may improve triaging of indeterminate adnexal masses or support earlier intervention^[25]^.

Artificial intelligence (AI) and machine-learning models are increasingly being used to integrate complex molecular data with clinical characteristics such as age, symptoms, and family history. Rather than focusing on the computational architecture, the clinical relevance lies in the ability of these models to identify subtle biomarker combinations that outperform traditional single-marker tests. Such tools may provide clinicians with risk scores or diagnostic probabilities that enhance early detection, reduce unnecessary procedures, and support personalized decision making^[20]^. Advances in methylation sequencing and chromatin accessibility profiling are revealing early epigenetic irregularities that occur well before overt tumor growth. Emerging assays are becoming more sensitive in detecting these early changes in blood-based samples. Since epigenetic alterations often precede structural genomic mutations, these techniques may offer some of the earliest measurable signals of ovarian carcinogenesis, positioning them as promising candidates for future screening platforms^[26]^.

**Figure 1.**
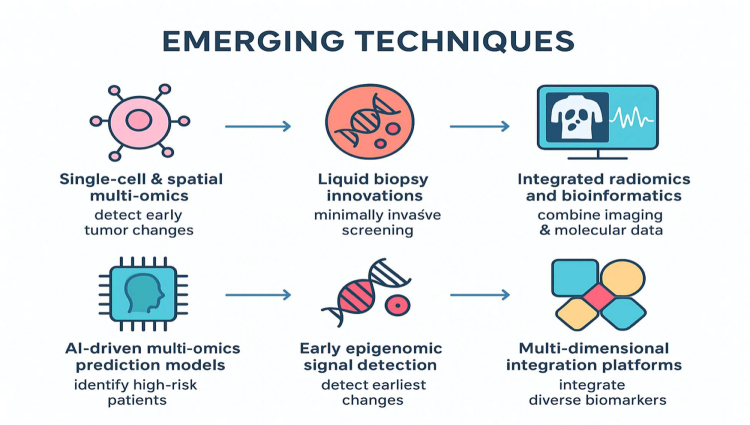
Emerging techniques.

New integrative bioinformatics platforms now combine genomic, transcriptomic, proteomic, metabolomic, and imaging data into unified patient profiles. These platforms help identify biomarker patterns that are consistently detectable across multiple biological layers, increasing their likelihood of clinical robustness. From a clinician’s perspective, such harmonized data may eventually evolve into simplified diagnostic panels or dashboards that support real-world decision making (Fig. 1)^[27]^.

### Applications and key studies

Integrative bioinformatics and machine learning techniques have been increasingly applied in ovarian cancer research to identify early detection biomarkers with enhanced sensitivity and specificity. These studies combine multi-omics datasets and clinical data to develop predictive models that can discriminate early-stage ovarian cancer from benign conditions or healthy controls^[28]^. One notable study by Sanches *et al* employed a multi-omics integration approach combining transcriptomics, proteomics, and metabolomics data from patient plasma samples. Using machine learning classifiers, including random forests and support vector machines, the authors identified a panel of biomarkers that improved early-stage ovarian cancer detection over cancer antigen 125 (CA125) alone, achieving an area under the ROC curve exceeding 0.90. Their approach highlighted the importance of integrating metabolite profiles with gene expression data to capture tumor-associated metabolic alterations^[29]^.

Another significant example is the work by Gonzalez Bosquet *et al*, who utilized a deep learning-based autoencoder model to analyze high-dimensional genomic and epigenomic data from early-stage ovarian tumors. Their model identified novel DNA methylation signatures that were highly predictive of disease presence. Importantly, the study incorporated explainable AI techniques to interpret feature contributions, facilitating clinical relevance and potential translation^[30]^. In a comprehensive meta-analysis, Wu and colleagues integrated publicly available transcriptomic datasets using network-based methods to identify gene modules associated with early ovarian cancer. Applying machine learning algorithms for feature selection and classification, they proposed a robust gene signature panel validated across independent cohorts. This study underscored the value of combining network biology with computational modeling for biomarker discovery^[31]^.

Liquid biopsy-based biomarker discovery has also benefited from integrative bioinformatics. For instance, Wang *et al* (2022) analyzed ctDNA and extracellular vesicle-derived RNA profiles using multi-modal integration and machine learning, revealing a biomarker signature capable of detecting ovarian cancer at early stages with high accuracy. This noninvasive approach holds promise for clinical screening applications^[7]^. These key studies collectively demonstrate the power of combining multi-omics data and advanced computational methods to overcome the limitations of traditional biomarkers. While promising, the translation of these findings into routine clinical diagnostics requires further validation, larger cohort studies, and standardized pipelines to ensure reproducibility and robustness.

### Challenges

Despite the growing promise of integrative bioinformatics for identifying early detection biomarkers in ovarian cancer, several important challenges continue to limit clinical translation. These obstacles arise not only from technical complexities but also from practical, biological, and implementation-related issues that directly influence the feasibility of introducing new biomarkers into routine care. The challenges in early detection of ovarian cancer are rooted in the biological complexities, including anatomical inaccessibility, nonspecific early symptoms, and pronounced molecular heterogeneity. These factors limit the effectiveness of conventional diagnostic tools and underscore the need for more sensitive and specific approaches. Integrative bioinformatics methods, as outlined in the Methods section, are specifically designed to address these obstacles by combining data from genomics, transcriptomics, proteomics, and metabolomics to capture subtle molecular changes indicative of early disease. For example, single-cell sequencing can resolve intra-tumoral heterogeneity, while liquid biopsy techniques allow noninvasive monitoring of ctDNA or RNA. By linking biological realities to computational strategies, these integrative approaches provide a pathway to overcome inherent diagnostic limitations and improve the detection of ovarian cancer at a stage when interventions are most likely to succeed.

#### Limited availability of early-stage samples

One of the most significant barriers is the scarcity of high-quality early-stage ovarian cancer samples. Because most patients present with advanced disease, the datasets available for studying early tumor biology remain small and heterogeneous. Clinically, this makes it difficult to validate biomarkers intended specifically for early detection, as they may perform well in research settings but struggle to demonstrate consistent accuracy in real-world populations^[32]^.

#### Biological heterogeneity of ovarian cancer

Ovarian cancer encompasses multiple histological subtypes, each with distinct molecular features. This biological heterogeneity complicates the identification of universal early detection markers. A biomarker that is effective for high-grade serous carcinoma may not apply to mucinous or endometrioid forms. For clinicians, this means that early detection strategies may need to be subtype-specific, adding complexity to screening, interpretation, and diagnostic workflows^[33]^.

#### Overfitting and limited generalizability

While sophisticated computational models can detect subtle molecular patterns, they are vulnerable to overfitting – performing well on the datasets used for model development but poorly on new patient populations. From a clinical perspective, this raises concerns about reliability, reproducibility, and the risk of false reassurance or unnecessary alarm. Biomarkers must demonstrate stability across diverse cohorts before being trusted in practice^[34]^.

#### Inconsistent data quality and lack of standardization

Multi-omics research often integrates data generated from different laboratories, platforms, and patient populations. Variation in sample handling, sequencing depth, biospecimen storage, and preprocessing methods can lead to inconsistent findings. For clinicians, this inconsistency can undermine confidence in emerging biomarkers and complicate efforts to compare results across studies^[12]^.

#### Challenges in multi-omics integration

Although integrating multiple molecular layers can strengthen biomarker robustness, combining diverse datasets remains analytically challenging. Misalignment across data types – or incomplete representation of certain omics layers – may obscure clinically relevant signals. Translating complex multi-omic relationships into practical clinical tests requires simplification, validation, and clear interpretation guidelines, all of which remain ongoing challenges^[35]^.

#### Limited prospective validation

Many promising biomarkers are discovered retrospectively. However, clinicians require prospective, longitudinal evidence to determine how these biomarkers behave in real time and across the natural course of disease development. Without such validation, biomarkers may lack sufficient accuracy for early detection, risk prediction, or screening^[36]^.

#### Cost, accessibility, and implementation barriers

Even when biomarkers show promise, the cost of multi-omics testing and the lack of standardized diagnostic pathways can delay clinical adoption. Resource-limited settings face additional barriers, including limited molecular testing infrastructure and workforce shortages. Ensuring accessibility is essential to prevent widening disparities in cancer detection^[37]^.

#### Ethical and communication considerations

Early detection raises unique ethical considerations, such as communicating risk, avoiding unnecessary anxiety, and managing indeterminate results. Integrating complex bioinformatics outputs into patient-centered discussions requires clear communication strategies that clinicians can effectively implement^[37]^.

### Clinical relevance and implications

The identification of early detection biomarkers in ovarian cancer through integrative bioinformatics has profound clinical implications. Early diagnosis is critical, as the prognosis of ovarian cancer is significantly better when detected at stage I or II compared to advanced stages. Integrative bioinformatics enables the discovery of biomarkers with enhanced specificity and sensitivity, potentially surpassing the limitations of conventional markers such as CA125 and transvaginal ultrasound^[7]^. By combining multi-omics data and computational modeling, bioinformatics approaches can identify panels of biomarkers rather than relying on single markers. These biomarker panels can improve the discrimination between benign and malignant ovarian lesions, reducing false positives and unnecessary interventions^[32]^.

Emerging biomarkers identified through bioinformatics, such as ctDNA, exosomal RNA, and proteomic signatures, can be detected in blood or other biofluids. This opens avenues for minimally invasive or liquid biopsy-based screening strategies, which are more acceptable to patients and allow repeated monitoring over time^[33]^. Integrative analysis can identify molecular signatures associated with high-risk subgroups. Patients with genetic predispositions (e.g., BRCA mutations) or specific epigenetic patterns may benefit from tailored surveillance programs, enabling precision medicine approaches in early detection^[34]^. Validated biomarkers can assist clinicians in triaging patients for diagnostic imaging, surgical evaluation, or early therapeutic interventions. Biomarker-driven decision making may reduce delays in diagnosis and optimize resource allocation in healthcare systems^[12]^.

Integrative bioinformatics provides a systematic framework for prioritizing biomarker candidates for preclinical and clinical validation. This accelerates the translation from computational discovery to clinical assays, bridging the gap between research and practice^[35,36]^. Ongoing advances in AI, single-cell sequencing, and longitudinal multi-omics studies are expected to refine early detection biomarker panels. In the long term, routine clinical implementation of bioinformatics-derived biomarkers could transform ovarian cancer screening, leading to earlier diagnoses, improved patient outcomes, and reduced mortality^[37–39]^.

## Conclusion

Early detection of ovarian cancer remains one of the most pressing challenges in gynecologic oncology, and integrative bioinformatics is rapidly emerging as a transformative tool with meaningful clinical promise. By harmonizing genomics, transcriptomics, proteomics, metabolomics, and imaging-derived biomarkers, these computational approaches allow for a more holistic understanding of disease biology and a more sensitive identification of early-stage molecular signals. Importantly, as emphasized throughout the revised manuscript, the growing alignment between bioinformatics methodologies and real-world clinical needs – robust sample preprocessing workflows, clinically interpretable quality-control metrics, and patient-centered thresholds for biomarker reliability – represents a major step toward translational feasibility.

Emerging techniques such as multi-modal machine learning, network-based biomarker discovery, and integrative risk-scoring systems are beginning to bridge the gap between high-dimensional research outputs and actionable bedside decisions. Nevertheless, challenges remain, including data heterogeneity, varying assay quality, limited validation across diverse populations, and regulatory barriers for clinical adoption. Addressing these issues will require stronger collaboration between computational scientists, clinicians, laboratory medicine specialists, and regulatory bodies to ensure that biomarker signatures are not only theoretically robust but also reproducible, explainable, and compatible with routine diagnostic workflows. Despite these hurdles, the trajectory of integrative bioinformatics is unmistakably toward greater clinical relevance. As datasets grow more representative and analytical tools become more transparent and clinician-friendly, the possibility of developing cost-effective, minimally invasive, and highly accurate early-detection biomarkers becomes increasingly achievable. Ultimately, the integration of advanced computational methods with clinically grounded research has the potential to transform ovarian cancer screening paradigms, enabling earlier diagnosis, more personalized risk assessment, and significantly improved patient outcomes.
